# Author Correction: ABHD5 inhibits YAP-induced c-Met overexpression and colon cancer cell stemness via suppressing YAP methylation

**DOI:** 10.1038/s41467-023-37521-w

**Published:** 2023-04-04

**Authors:** Yan Gu, Yanrong Chen, Lai Wei, Shuang Wu, Kaicheng Shen, Chengxiang Liu, Yan Dong, Yang Zhao, Yue Zhang, Chi Zhang, Wenling Zheng, Jiangyi He, Yunlong Wang, Yifei Li, Xiaoxin Zhao, Hongwei Wang, Jun Tan, Liting Wang, Qi Zhou, Ganfeng Xie, Houjie Liang, Juanjuan Ou

**Affiliations:** 1 Department of Oncology and Southwest Cancer Center, Southwest Hospital, Third Military Medical University (Army Medical University), 400038 Chongqing, China; 2 Biomedical Analysis Center, Third Military Medical University (Army Medical University), 400038 Chongqing, China; 3 Department of Oncology, Fuling Central Hospital of Chongqing City, 408000 Chongqing, China

**Keywords:** Targeted therapies, Colon cancer

Correction to: *Nature Communications* 10.1038/s41467-021-26967-5, published online 18 November 2021

This Article contains an error in Figure 2j. A higher magnification of the shCTRL (Savolitinib) panel was inserted by mistake in the panel shCTRL (Vehicle).

The correct version of figure 2j is shown below.


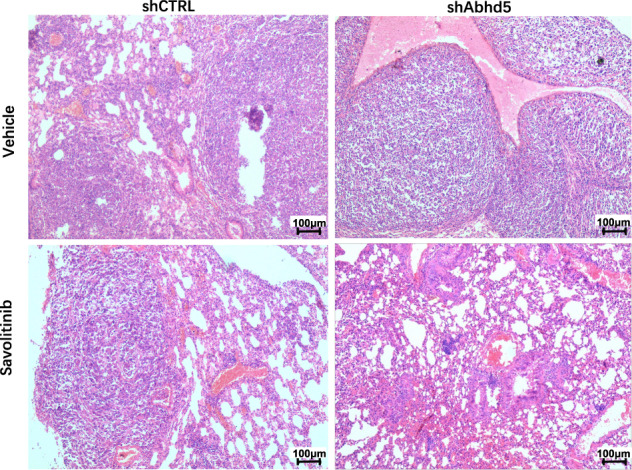


This error has been corrected in the PDF or HTML versions of the Article.

