# Supplementary material for: ABHD5 inhibits YAP-induced c-Met overexpression and colon cancer cell stemness via suppressing YAP methylation
Source: Nat Commun. 2021 Nov 18;12:6711. doi: 10.1038/s41467-021-26967-5 (PMC8602706; doi:10.1038/s41467-021-26967-5)
Supplement: Supplementary file 1 — Supplementary Information [file 41467_2021_26967_MOESM1_ESM.pdf]

ABHD5 inhibits YAP-induced c-Met overexpression and colon cancer cell stemness via suppressing YAP methylation

Yan Gu, Yanrong Chen, Lai Wei, Shuang Wu, Kaicheng Shen, Chengxiang Liu, Yan Dong, Yang Zhao, Yue Zhang, Chi Zhang, Wenling Zheng, Jiangyi He, Yunlong Wang, Yifei Li, Xiaoxin Zhao, Hongwei Wang, Jun Tan, Liting Wang, Qi Zhou, Ganfeng Xie, Houjie Liang and Juanjuan Ou

Supplementary materials

Antibodies	Source	Identifier
ABHD5	NOVUS	H00051099-M01,RRID:AB_536536 (for WB,IHC)
ABHD5	SIGMA	HPA035851,RRID:AB_10671942 (for IF)
β-Actin	EarthOx	E021070-01
c-Met	CST	8198,RRID:AB_10858224 (for WB)
c-Met	Santa	SC-8057,RRID:AB_673755 (for IHC)
GAPDH	Proteintech	60004-1-Ig,RRID:AB_2107436
β-Catenin	Santa	SC-393501 (IHC)
β-Catenin	CST	8480,RRID:AB_11127855 (for WB,IF)
TCF4	Santa	SC-166699,RRID:AB_2199823(for WB)
TCF4	CST	2569,RRID:AB_2199816 (for CHIP)
YAP1	NOVUS	NB110-58358,RRID:AB_922796 (for WB,IF and CHIP)
YAP1	Santa	SC-101199,RRID:AB_1131430 (for IHC)
YAP1 (SU33-06)	NOVUS	NBP2-67467(for IP)
H2A	NOVUS	NB100-56346,RRID:AB_838346
TEAD1	Abcam	Ab133533,RRID:AB_2737294
Tubulin	Proteintech	HRP-66031,RRID:AB_2687491
GFP	NOVUS	NB600-308,RRID:AB_10003058
YAP km342	College of Life Science and Technology of Tongji University	
p-YAP(S127)	Abcam	Ab205270,RRID:AB_2813833
CRM1(A-7)	Santa	SC-374124,RRID:AB_10917075
ATGL(PNPLA2)	CST	2439
CD44	CST	3570,RRID:AB_2076465

C-Jun	CST	9165
CyclinD1	CST	2978
DPY30	NOVUS	45110002,RRID:AB_10702185 (for WB)
DPY30	NOVUS	NBP2-32201 (for IHC)
Ubiquitin	Abcam	Ab134953,RRID:AB_2801561
LPAR1	Abcam	Ab23698,RRID:AB_447619
The Alexa Fluor 594 anti-mouse IgG	Thermo Fisher	A11037
The Alexa Fluor 647 anti-mouse IgG	Santa	sc-516609
The FITC anti-rabbit IgG	EarthOx	E031220
The dylight 649 anti-Rabbit IgG(H+L)	EarthOx	E032620
Anti-histone H3(trimethyl K4)	Abcam	Ab8580,RRID:AB_306649(for Chip, WB)
Anti-hSET1/SET	Abcam	Ab70378,RRID:AB_1951955(for Chip,WB)
SETD1A	Novus	NB100-558,RRID:AB_2185760(for IP, WB and Chip)
PE/Cy7 anti-mouse/human CD44	Biolegend	103029
APC anti-Human CD133	BD	566596
Lgr5/GPR49 Antibody	novus	NLS1236,, RRID:AB_10001136

**Critical Commercial Assays**	**Source**	**Identifier**
ABHD5 protein	NOVUS	H00051099-P01,(for Protein-protein interaction experiment based on HuprotTM human protein microarray assay)
Colon cancer tissue microarray	SHANGHAI OUTDO BIOTECH CO.,LTD.	HColA180Su16
Colon cancer tissue microarray	SHANGHAI OUTDO BIOTECH CO.,LTD.	HColA180Su13
Cell Counting Kit-8	Dojindo	CK04
Dynabeads Protein G Immunoprecipitation Kit	Thermo Fisher	10007D
Human lysophosphatidic acid (LPA) Elisa kit	CUSABIO	CSB-EQ028005HU
Plasmid Midi kit	OMEGA	D6904-03
SYBR Premix Ex Taq	Takara	RR420
Puromycin Dihydrochloride	Beyotime	ST551
Pierce Protease Inhibitor Mini Tablets	Roche	4693159001
Phosphatase Inhibitor Mini Tablets	Roche	4906837001
Dual-Luciferase® Reporter Assay System	Promega	E1910
Nucleus and Cytoplasmic Extraction Reagent Kit	Beyotime	P0027
ALDEFLUOR Stem Cell Identification kit	Stemcell	1700

Cells	Source	Identifier
HCT 116	ATCC	CCL-247, RRID:CVCL_0291
SW620	ATCC	CCL-227, RRID:CVCL_0547
RKO	ATCC	CRL-2577, RRID:CVCL_0504
MC38	Fuheng	FH0125,
CT26	ATCC	CRL-2638, RRID:CVCL_7256

Primers	Source	Identifier
GAPDH qPCR Forward primer: GAGCGAGATCCCTCCAAAAT	N/A	This paper
GAPDH qPCR For Reverse primer: GGCTGTTGTCATACTTCTCATGG	N/A	This paper
Abhd5 qPCR Forward primer: CAGCATCCAGTCCTTACGACCA	N/A	This paper
Abhd5 qPCR Reverse primer: GTTCAGTCCACAGTGTCGCAGA	N/A	This paper
DPY30 qPCR Forward primer: AGAAAAGTCATCAAAGCAGAAGGTAG	N/A	This paper
DPY30 qPCR Reverse primer: CAGGTAGGCACGAGTTGGC	N/A	This paper
MET qPCR Forward primer: TGCACAGTTGGTCCTGCCATGA	N/A	This paper
MET qPCR Reverse primer: CAGCCATAGGACCGTATTTCGG	N/A	This paper
CTGF qPCR Forward primer: CTTGCGAAGCTGACCTGGAAGA	N/A	This paper
CTGF qPCR Reverse primer: CCGTCGGTACATACTCCACAGA	N/A	This paper
DKK1 qPCR Forward primer: GGTATTCCAGAAGAACCACCTTG	N/A	This paper
DKK1 qPCR Reverse primer: CTTGGACCAGAAGTGTCTAGCAC	N/A	This paper
ITGB2 qPCR Forward primer: AGTCACCTACGACTCCTTCTGC	N/A	This paper
ITGB2 qPCR Reverse primer: CAAACGACTGCTCCTGGATGCA	N/A	This paper
Birc5 qPCR Forward primer: CCACTGAGAACGAGCCAGACTT	N/A	This paper
Birc5 qPCR Reverse Forward primer: GTATTACAGGCGTAAGCCACCG	N/A	This paper

AREG qPCR Forward primer: GCACCTGGAAGCAGTAACATGC	N/A	This paper
AREG qPCR Reverse primer: GGCAGCTATGGCTGCTAATGCA	N/A	This paper

**Plasmids**	**Source**	**Identifier**
SMYD1 human shRNA plasmid	Origen	TL301486
SMYD2 human shRNA plasmid	Origen	TL301485

**Oligonucleotides**	**Source**	**Identifier**
h. shRNA targeting sequence: *YAP*: GCTTTGAGTTCTGACATCCUU	N/A	This paper
h. shRNA targeting sequence: *SET1A:* GGAAAGAGCCATCGGAAATTT	N/A	This paper
h. siRNA targeting sequence: *WDR5*: UAAACAAGGUUAUCCUCUGUU	N/A	This paper
h. siRNA targeting sequence: *RbBP5*: CAGGTGTCTCTCAACAAGCTAUU	N/A	This paper
h. siRNA targeting sequence: *ASH2L*: GGGAGGAAGAGCCGGGUUUTT	N/A	This paper
h. siRNA targeting sequence: *DPY30*: ACAACGUUGAGAGAAUUU	N/A	This paper
h. shRNA targeting sequence: A*BHD5 1#*: TCTTTGCACCAACAGACCTGTCTATGCTT	N/A	This paper
h. shRNA targeting sequence: A*BHD5 2#*: AGACGATACTGTGACAGAATACATCTACC	N/A	This paper
m. shRNA targeting sequence: *Abhd5*: GCAGCATTGACTCCCTTTAAC	N/A	This paper
h. shRNA targeting sequence: *MET*: GCACGACAAATACGTTGAAAT	N/A	This paper
h. shRNA targeting sequence: *SMYD3*: GCATTAAAGCAGCGTATC	N/A	This paper
h. shRNA targeting sequence: *SMYD4*: AAAGGATCTACGCATAAGC	N/A	This paper
h. shRNA targeting sequence: *SMYD5*: TTATGGACAATTTCCTCCT	N/A	This paper
h. shRNA targeting sequence: *SET1B*: TATCATCTCGGTGATCCTC	N/A	This paper

h. shRNA targeting sequence: *SET7*: ATAAATTTCCCATAAAGTG	N/A	This paper
h. shRNA targeting sequence: *Suv39H1*: TGTAATCAAAGGTGAGCTC	N/A	This paper
h. shRNA targeting sequence: *Suv39H2*: TAAGTACACATATACTAAG	N/A	This paper
h. shRNA targeting sequence: *EZH2*: TATCATCTCGGTGATCCTC	N/A	This paper
h. shRNA targeting sequence: *DPY30*: ACAACGUUGAGAGAAUUU	N/A	This paper

**Software and Algorithms**		
Prism 8	GraphPad	www.graphpad.com/scientific software/prism
ImageJ v1.8.0	National Institutes of Health	imagej.nih.gov/ij
L-Calc	Stem Cell Technologies	www.stemcell.com/tutorials/lcsetup.exe
FACSDiva v8.0.1	Bekton Dikinson	www.bdbiosciences.com/

Supplementary figure legend

Supplementary Fig. 1 ABHD5 suppresses the self-renewal capacity of CRC cells.

a, Immunoblotting of ABHD5 in control (shCTRL) and ABHD5-knockdown (shABHD5) HCT116 cells using a second shRNA (shRNA2).

b, c Sphere formation assay of control (shCTRL) and ABHD5-knockdown (shABHD5) HCT116 cells using a second shRNA (shRNA2). The representative pictures of spheres are shown **(b)**. The number and the size (n=5, 10 biologically independent samples) of the spheres were quantified **(c)**. Scale bar, 50μm. AU, arbitrary units.

d, e Sphere formation assay of control (shCTRL) and ABHD5-knockdown (shABHD5) SW620 cells. The representative pictures of spheres are shown **(d)**. The number and the size (n=5, 10 biologically independent samples) of the spheres were quantified **(e)**. Scale

bar, 50μm. AU, arbitrary units.

f, Sphere formation assay of control (CTRL) and *ABHD5*-overexpression (ABHD5) HCT116 cells. The number and the size (n=5, 10 biologically independent samples) of the spheres were quantified. AU, arbitrary units.

g, h AI growth in soft agar by shCTRL and shABHD5 HCT116 cells using a second shRNA (shRNA2). Photomicrographs were taken after staining with iodinitrotetrazolium violet (4mg/ml) of soft agar colonies grown for 14 days **(g)**. The number and the size (n=5, 10 biologically independent samples) of the colonies were quantified **(h).**

i, Stem cell frequencies of control (CTRL) and *ABHD5*-overexpressing (ABHD5) HCT116 cells were determined using L-Calc software. The upper table shows the number of tumors with a positive response (response = tumor > 0.1 cm^3 at 20 days post-injection)/total number of tumors and is depicted in the pie chart diagram below. The lower table shows stem cell frequency ± 95% confidence intervals.

j, The analyses of the relative primary and second sphere-forming unit of ABHD5 overexpressing HCT116 cells (ABHD5) and control cells (CTRL) digested from the xenografts derived from the indicated injected cell number. (n=3 biologically independent samples).

(Data are shown as mean ± s.e.m.. Unpaired two-sided Student's t test was used in panels **c** (right), **e**(right), **f**(right) and **h,** and panels **c** (left), **e** (left), **f**(right) and **j** were analyzed using two way ANOVA and Sidak's multiple comparison test. Source data are provided as a Source Data file.)

Supplementary Fig. 2 Loss of ABHD5 activates c-Met without affecting β-Catenin.

a, Subcellular fractionation immunoblotting of cytoplasmic and nuclear β-Catenin in shCTRL or shABHD5 HCT116 cells (*ABHD5* knockdown by shRNA2) treated with either Wnt3a (250ng/ml) or vehicle (PBS).

b, Immunoblotting of ABHD5, c-Met and β-Catenin expression levels in the intestine tumors and intestine mucosa of $Apc^{Min/+}$/$Abhd5^{+/+/Cre+}$ and $Apc^{Min/+}$/$Abhd5^{f/f/Cre+}$ male mice at 100 days of age.

c, Representative immunostaining images of β-Catenin in the intestine tumors and intestine mucosa of $Apc^{Min/+}$/$Abhd5^{+/+/Cre+}$ and $Apc^{Min/+}$/$Abhd5^{f/f/Cre+}$ male mice at 100 days of age. Scale bar, 500 μm and 200 μm.

d, Immunoblotting of ABHD5 and c-Met expression levels in shCTRL or shABHD5 HCT116 cells (*ABHD5* knockdown by shRNA2) treated with either XAV-939 (10μM) or vehicle (DMSO).

Supplementary Fig. 3 Loss of ABHD5 also activates c-Met expression in β-Catenin inactive colon cancer cell line RKO.

a, Immunoblotting of ABHD5 and c-Met expression levels in shCTRL or shABHD5 RKO cells.

b, Sphere formation assay of shCTRL or shABHD5 RKO cells transfected with either the control shRNA or *MET*-knockdown shRNA (*MET* KD). The representative pictures of

spheres in shCTRL group and shABHD5 group are shown (left, scale bar = 20μm), and the number of spheres was quantified (right). (n = 3 biologically independent samples)

c, Anchorage-independent growth assay of shCTRL or shABHD5 RKO cells transfected with either the control shRNA or *MET*-knockdown shRNA (*MET* KD). The representative pictures of spheres in shCTRL group and shABHD5 group are shown (left), and the number of colonies was quantified (right). (n = 3 biologically independent samples)

(Data are shown as mean ± s.e.m.. Panels **b-c** were analyzed using two way ANOVA and Sidak's multiple comparison test.Source data are provided as a Source Data file. Source data are provided as a Source Data file.)

Supplementary Fig. 4 YAP inhibitor vertiporfin efficiently suppresses the stemness of ABHD5 deficient CRC cells.

a, Statistical analyses of sphere formation assay of shCTRL or shABHD5 HCT116 cells treated with either the vehicle (DMSO) or YAP inhibitor verteporfin (15 μM). (n = 3 biologically independent samples)

b, Statistical analyses of anchorage-independent growth assay of shCTRL or shABHD5 HCT116 cells treated with either the vehicle (DMSO) or verteporfin (15 μM). (n = 3 biologically independent samples)

c, Statistical analyses of the subcutaneous tumour volume of shCTRL or shABHD5 HCT116 cell-derived xenografts in the indicated groups after 21 days administration of verteporfin. Control or *ABHD5* knockdown HCT116 cells were injected into NOD-SCID

mice subcutaneously, and the mice were treated with vehicle control (8% DMSO 100 μl) or 30 mg/kg verteporfin (i.p.) once daily for 21 days when tumors reached 120-275 mm^3. (n = 6 mice/group)

(Data are shown as mean ± s.e.m.. All data were analyzed using two way ANOVA and Sidak's multiple comparison test. Source data are provided as a Source Data file.)

Supplementary Fig. 5 YAP-MET signalling regulated by ABHD5 is conserved between murine and human CRCs.

a, Immunoblotting of ABHD5 and c-Met expression levels in shCTRL or shAbhd5 murine colon cancer cell line CT26 cells treated with *Yap*-knockdown or verteporfin (10 μM).

b, Immunoblotting of ABHD5 and c-Met expression levels in shCTRL or shAbhd5 murine colon cancer cell line MC38 cells treated with *Yap*-knockdown or verteporfin (10 μM).

c, Statistical analyses of sphere formation assay and anchorage-independent growth assay of shCTRL or shAbhd5 CT26 cells transfected with either the control shRNA or *Yap*-knockdown shRNA (*Yap* KD). (n = 3 biologically independent samples)

d, Statistical analyses of sphere formation assay and anchorage-independent growth assay of shCTRL or shAbhd5 MC-38 cells transfected with either the control shRNA or *Yap*-knockdown shRNA (*Yap* KD). (n = 3 biologically independent samples)

(Data are shown as mean ± s.e.m.. Panels **c** and **d** were analyzed using two way ANOVA and Sidak's multiple comparison test. Source data are provided as a Source Data file.)

Supplementary Fig. 6 ABHD5 deficiency promotes YAP methylation at K342 in colon cancer cells.

a, Immunoblotting of the expression levels of YAP in shCTRL and shABHD5 HCT116 cells (ABHD5 knockdown by shRNA2).

b, Subcellular fractionation immunoblotting of the expression levels of the indicated proteins in shCTRL and shABHD5 HCT116 cells (ABHD5 knockdown by shRNA2).

c, Immunoblotting of the expression levels of the indicated proteins in shCTRL and shABHD5 SW620 cells.

Supplementary Fig. 7. ABHD5 regulates SET1A-induced chromatin accessibility to synergistically control YAP-induced c-Met transcription.

a, Immunoblotting of H3K4me3 expression levels in shCTRL or shABHD5 HCT116 cells.

b, ATAC assay showing the shift of chromatin accessibility between shCTRL and shABHD5 HCT116 cells.

c, Multiomics view of Hi-C, ATAC-seq, ChIP-seq and RNA-seq at the locus of *MET*. The region of *MET* where 3D chromatin phenotypes are correlated with epigenomic and transcriptomic phenotypes. Triangular heatmaps from top to bottom: Hi-C contact heatmaps of *MET* region from shCTRL or shABHD5 HCT116 cells. The tracks below showing the shift of c-Met transcription levels, ChIP-qPCR confirmation of H3K4me3 ChIP fragment enrichment, chromatin accessibility and ChIP-qPCR confirmation of

SET1A ChIP fragment enrichment of *MET* between shCTRL and shABHD5 HCT116 cells.

Supplementary Fig. 8 ABHD5 regulates YAP methylation independent of its lipolytic activity.

a, Immunoblotting of ABHD5 and PNPLA2 expression levels in shCTRL and shABHD5 HCT116 cells.

b, Immunoblotting of β-Catenin target gene expression levels in shCTRL and shPNPLA2 HCT116 cells.

c, Representative immunofluorescent staining of β-Catenin and YAP in shCTRL and shPNPLA2 HCT116 cells. (Scale bar, 25μm)

d, Subcellular fractionation immunoblotting of cytoplasmic and nuclear β-Catenin and YAP in shCTRL and shPNPLA2 HCT116 cells treated with either Wnt3a (250ng/ml), EGF (200ng/ml) or vehicle (PBS).

e, β-Catenin and YAP immune complexes were immunoprecipitated from shCTRL and shPNPLA2 HCT116 cells and subjected to immunoblotting of TCF4 and TEAD1.

f, g, Top-flash (Wnt/β-Catenin pathway-responsive firefly luciferase plasmid) reporter gene assay **(f)** and TEAD luciferase reporter assay **(g)** in shCTRL and shPNPLA2 HCT116 cells. (n = 3 biologically independent samples)

h, Immunoblotting of the expression levels of the indicated proteins in shCTRL and shABHD5 PNPLA2 null HCT116 cells.

i, TEAD luciferase reporter assay in shCTRL and shABHD5 PNPLA2 null HCT116 cells. (n = 3 biologically independent samples)

j, Immunoblotting of YAP K342me and c-Met expression levels in control and ABHD5 null HCT116 cells transfected with *ABHD5* mutants (Q130P, E260K) or control vectors.

k, TEAD luciferase reporter assay in control and ABHD5 null HCT116 cells transfected with *ABHD5* mutants (Q130P, E260K) or control vectors. (n = 3 biologically independent samples)

(Data are shown as mean ± s.e.m.. Unpaired two-sided Student's t test was used in panels **f**, **g** and **i**, whereas panel **k** was analyzed using two way ANOVA and Sidak's multiple comparison test. Source data are provided as a Source Data file.)

Supplementary Fig. 9 LPA signaling is not the predominant pathway attributable to ABHD5 deficiency-induced YAP methylation.

a, ELISA assay of LPA concentration in the supernatant of shCTRL and shABHD5 HCT116 cells. (n = 3 biologically independent samples)

b, Immunoblotting of LPA receptor 1 (LPAR1) expression levels in shCTRL and shABHD5 HCT116 cells.

c, Immunoblotting of nuclear YAP expression levels in shCTRL and shABHD5 HCT116 cells treated with LPA receptor inhibitor Ki16425 (10 μM) or vehicle (DMSO).

d, TEAD luciferase reporter assay in shCTRL and shABHD5 HCT116 cells treated with Ki16425 (10 μM) or vehicle (DMSO). (n = 3 biologically independent samples)

e, Immunoblotting of c-Met expression levels in shCTRL and shABHD5 HCT116 cells treated with Ki16425 (10 μM) or vehicle (DMSO).

f, The statistical analyses of sphere formation assay and anchorage-independent growth assay of shCTRL and shABHD5 HCT116 cells treated with Ki16425 (10 μM) or vehicle (DMSO). (n = 3 biologically independent samples)

(Data are shown as mean ± s.e.m.. Unpaired two-sided Student's t test was used in panel **a**, whereas panels **d** and **f** were analyzed using two way ANOVA and Sidak's multiple comparison test. Source data are provided as a Source Data file.)

Supplementary Fig. 10 DPY30 mediates ABHD5 deficiency-induced stemness in CRCs.

a, Immunoblotting of ABHD5 and DPY30 expression levels in shCTRL and shABHD5 HCT116 cells (ABHD5 knockdown by shRNA2).

b, Immunoblotting of DPY30 expression levels in shCTRL and shABHD5 SW620 cells.

c, The statistical analyses of sphere formation assay and anchorage-independent growth assay of shCTRL and sh*ABHD5* HCT116 cells transfected with the control shRNA or *DPY30*-knockdown shRNA (*DPY30* KD). (n = 3 biologically independent samples)

d, The shCTRL and shABHD5 HCT116 cells were transfected with the control shRNA or *DPY30*-knockdown shRNA (*DPY30* KD), and the transfected cells were injected into the NSG mice to establish subcutaneous xenografts. The tumor volume of the subcutaneous

xenografts were analyzed. (n = 6 mice/group)

e, Subcellular fractionation immunoblotting of cytoplasmic and nuclear DPY30 in shCTRL or shABHD5 HCT116 cells (ABHD5 knockdown by shRNA2). H2A as a loading control of nucleic protein.

(Data are shown as mean ± s.e.m.. Panels **c** and **d** were analyzed using two way ANOVA and Sidak's multiple comparison test. Source data are provided as a Source Data file.)

Supplementary Fig. 11 ABHD5 inhibits the nuclear translocation of DPY30 to suppress histone methylation and synergistically impede YAP-induced c-Met activation.

a, Gene ontology (GO: cellular compartment) analysis of differentially expressed genes immunoprecipitated by DPY30 antibody between control and *ABHD5*-knockdown HCT116 cells.

b, Gene ontology (GO: biological process) analysis of differentially expressed genes immunoprecipitated by DPY30 antibody between control and *ABHD5*-knockdown HCT116 cells.

Fig S1

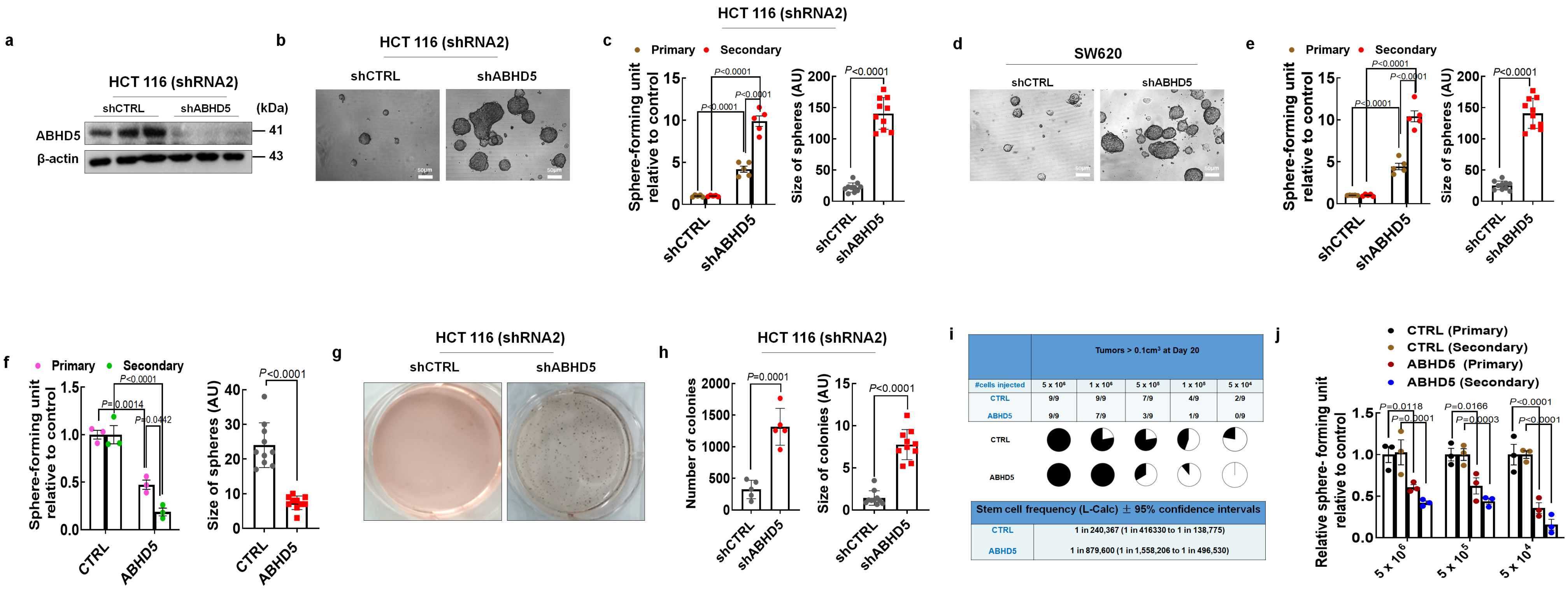

a
HCT 116 (shRNA2)
shCTRL
shABHD5
(kDa)
ABHD5
β-actin
41
43
b
HCT 116 (shRNA2)
shCTRL
shABHD5
c
HCT 116 (shRNA2)
Primary
Secondary
Sphere-forming unit relative to control
Size of spheres (AU)
P<0.0001
shCTRL
shABHD5
d
SW620
shCTRL
shABHD5
e
Primary
Secondary
Sphere-forming unit relative to control
Size of spheres (AU)
P<0.0001
shCTRL
shABHD5
f
Primary
Secondary
Sphere-forming unit relative to control
P<0.0001
P=0.0014
P=0.0442
Size of spheres (AU)
CTRL
ABHD5
g
HCT 116 (shRNA2)
shCTRL
shABHD5
h
HCT 116 (shRNA2)
Number of colonies
P=0.0001
Size of colonies (AU)
P<0.0001
shCTRL
shABHD5
i
Tumors > 0.1cm³ at Day 20
#cells injected | 5 x 10⁶ | 1 x 10⁶ | 5 x 10⁵ | 1 x 10⁵ | 5 x 10⁴
CTRL | 9/9 | 9/9 | 7/9 | 4/9 | 2/9
ABHD5 | 9/9 | 7/9 | 3/9 | 1/9 | 0/9
CTRL
ABHD5
Stem cell frequency (L-Calc) ± 95% confidence intervals
CTRL 1 in 240,367 (1 in 416330 to 1 in 138,775)
ABHD5 1 in 879,600 (1 in 1,558,206 to 1 in 496,530)
j
CTRL (Primary)
CTRL (Secondary)
ABHD5 (Primary)
ABHD5 (Secondary)
Relative sphere- forming unit relative to control
P=0.0118
P=0.0001
P=0.0166
P=0.0003
P<0.0001
P<0.0001
5 x 10⁶
5 x 10⁵
5 x 10⁴

Fig S2

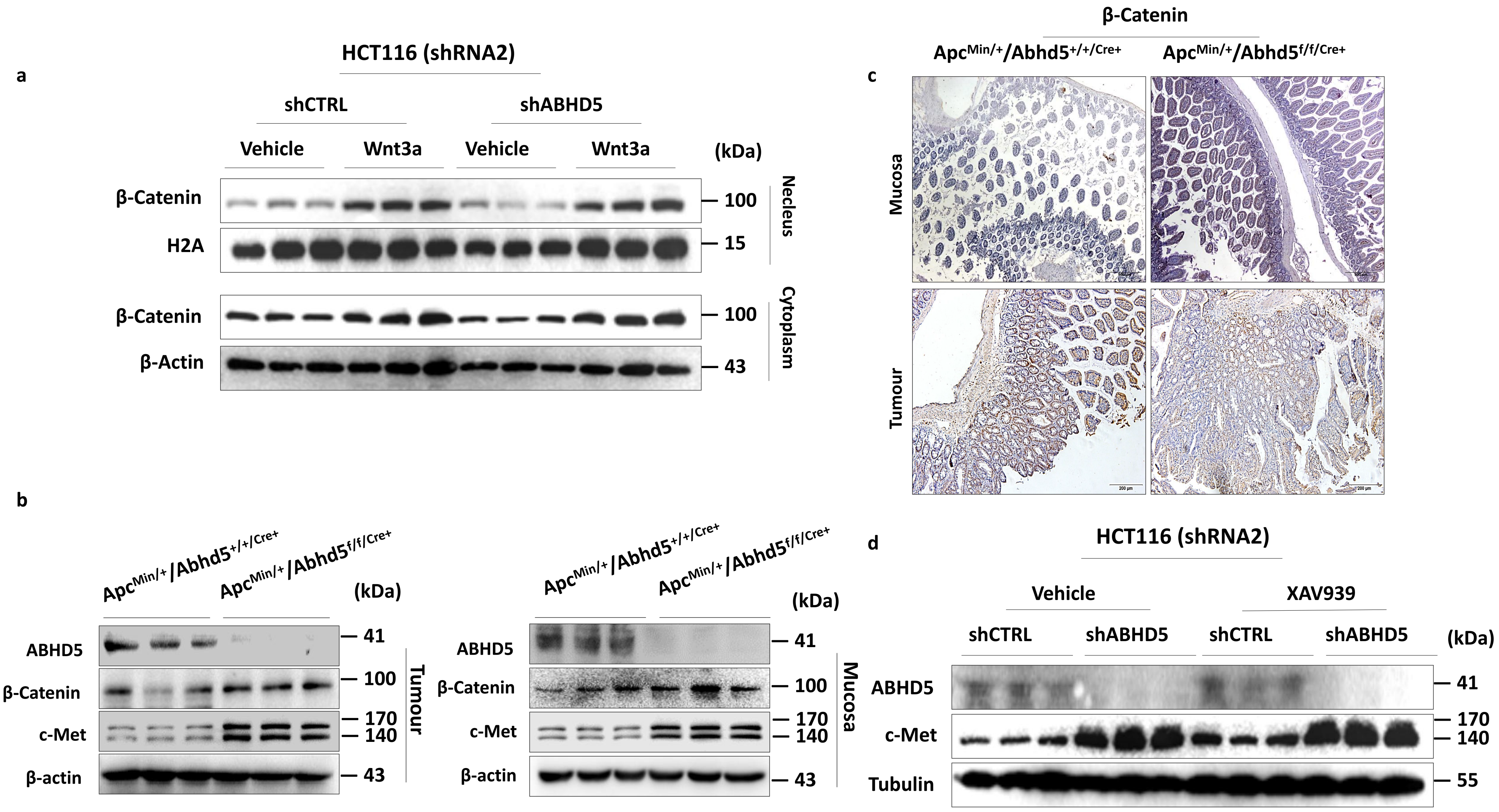
a
HCT116 (shRNA2)
shCTRL
shABHD5
Vehicle
Wnt3a
Vehicle
Wnt3a
(kDa)
β-Catenin
H2A
β-Catenin
β-Actin
100
15
100
43
Necleus
Cytoplasm
b
$Apc^{Min/+}/Abhd5^{+/+/Cre+}$
$Apc^{Min/+}/Abhd5^{f/f/Cre+}$
(kDa)
ABHD5
β-Catenin
c-Met
β-actin
41
100
170
140
43
Tumour
$Apc^{Min/+}/Abhd5^{+/+/Cre+}$
$Apc^{Min/+}/Abhd5^{f/f/Cre+}$
(kDa)
ABHD5
β-Catenin
c-Met
β-actin
41
100
170
140
43
Mucosa
c
β-Catenin
$Apc^{Min/+}/Abhd5^{+/+/Cre+}$
$Apc^{Min/+}/Abhd5^{f/f/Cre+}$
Mucosa
Tumour
d
HCT116 (shRNA2)
Vehicle
XAV939
shCTRL
shABHD5
shCTRL
shABHD5
(kDa)
ABHD5
c-Met
Tubulin
41
170
140
55

Fig S3

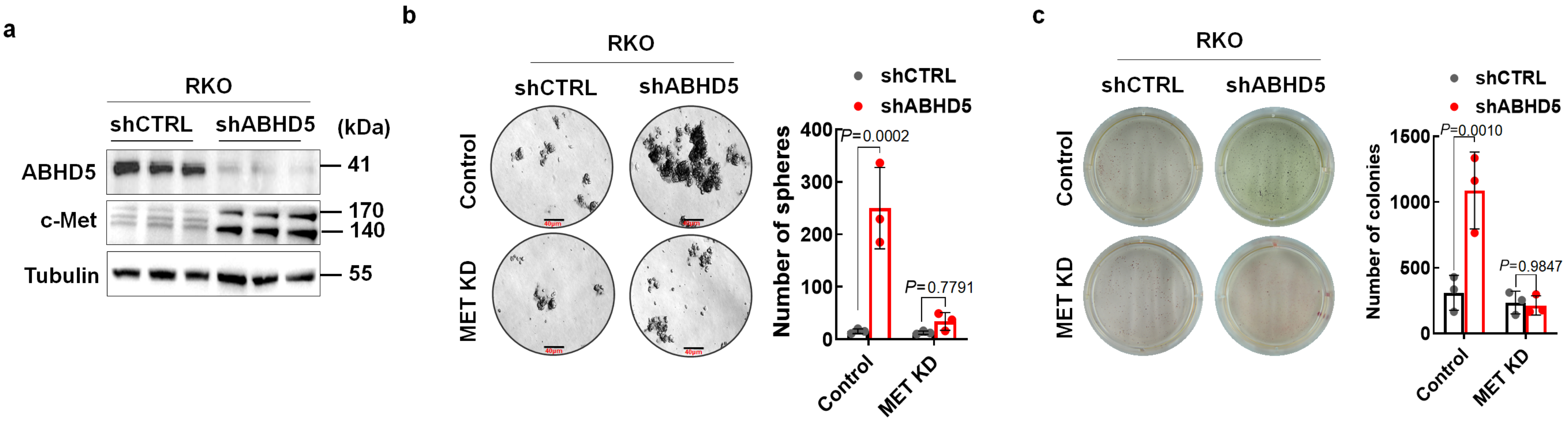
a
RKO
shCTRL
shABHD5
(kDa)
ABHD5
41
c-Met
170
140
Tubulin
55
b
RKO
shCTRL
shABHD5
Control
MET KD
shCTRL
shABHD5
Number of spheres
P=0.0002
P=0.7791
400
300
200
100
0
Control
MET KD
c
RKO
shCTRL
shABHD5
Control
MET KD
shCTRL
shABHD5
Number of colonies
P=0.0010
P=0.9847
1500
1000
500
0
Control
MET KD

Fig S4

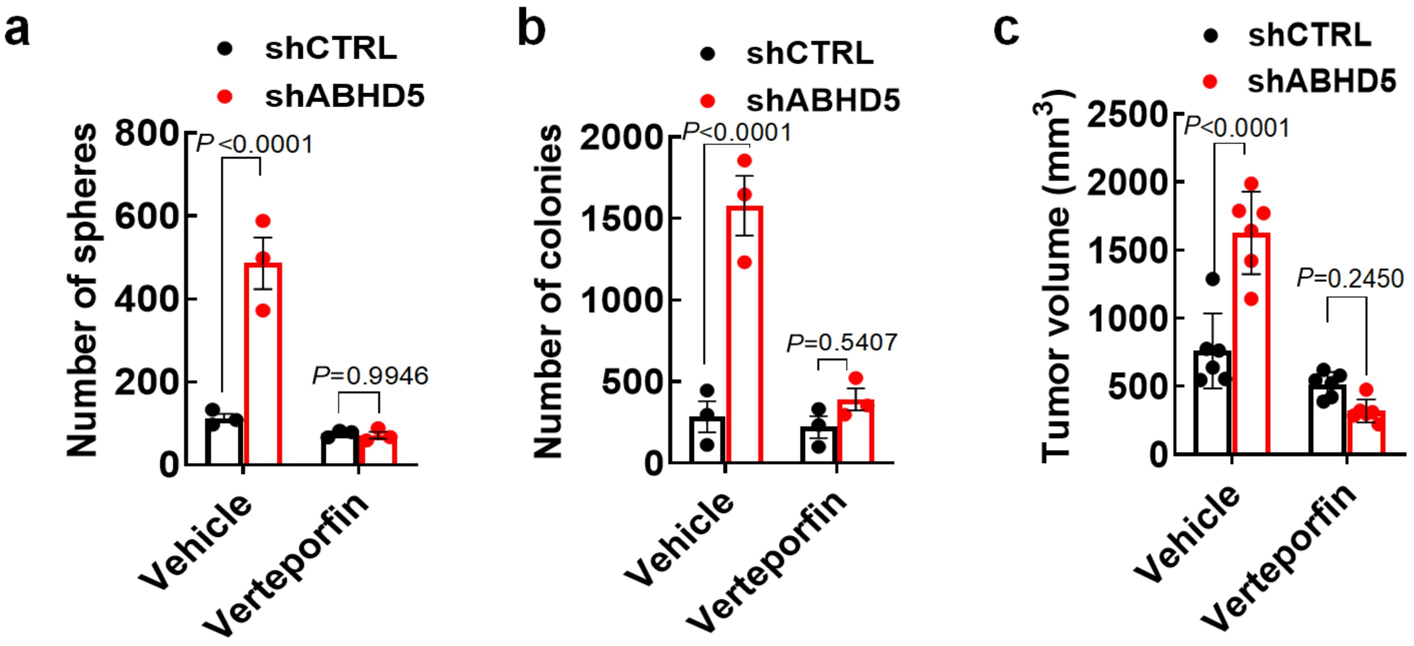

a
shCTRL
shABHD5
Number of spheres
800
600
400
200
0
P<0.0001
P=0.9946
Vehicle
Verteporfin
b
shCTRL
shABHD5
Number of colonies
2000
1500
1000
500
0
P<0.0001
P=0.5407
Vehicle
Verteporfin
c
shCTRL
shABHD5
Tumor volume (mm3)
2500
2000
1500
1000
500
0
P<0.0001
P=0.2450
Vehicle
Verteporfin

Fig S5

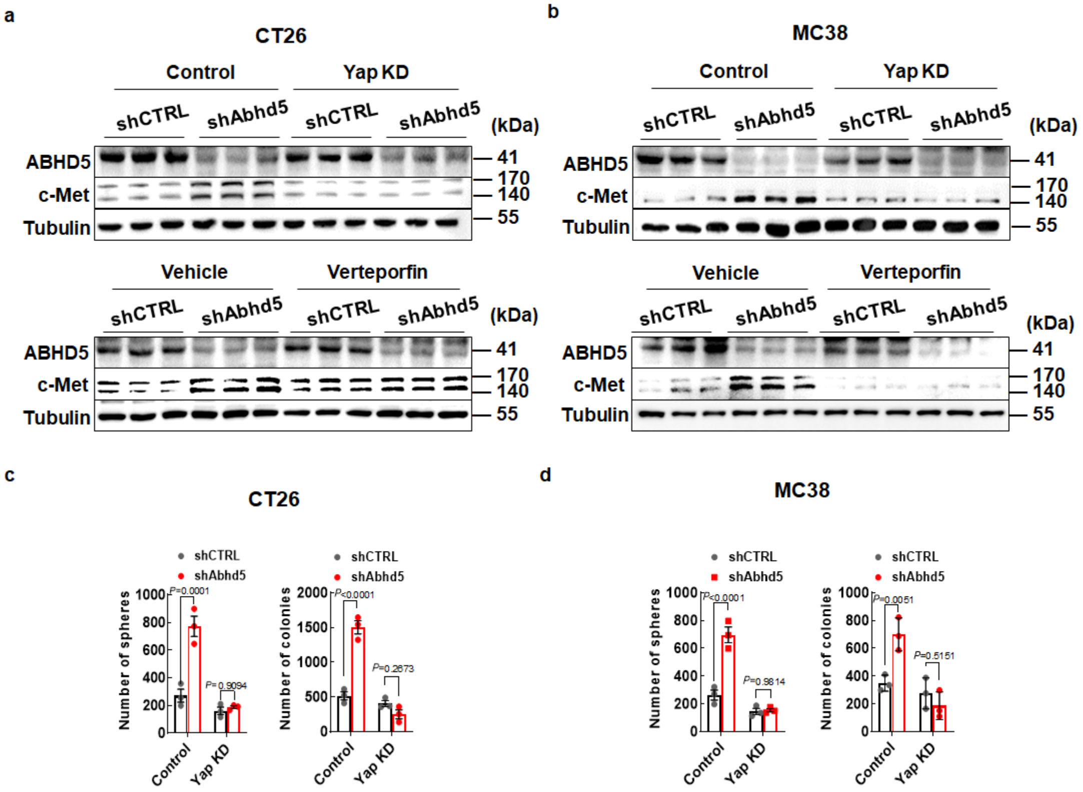
a
CT26
Control
Yap KD
shCTRL
shAbhd5
(kDa)
ABHD5
c-Met
Tubulin
41
170
140
55
Vehicle
Verteporfin
b
MC38
c
d
Number of spheres
Number of colonies
Control
Yap KD
P=0.0001
P=0.9094
P<0.0001
P=0.2673
P<0.0001
P=0.9814
P=0.0051
P=0.5151

Fig S6

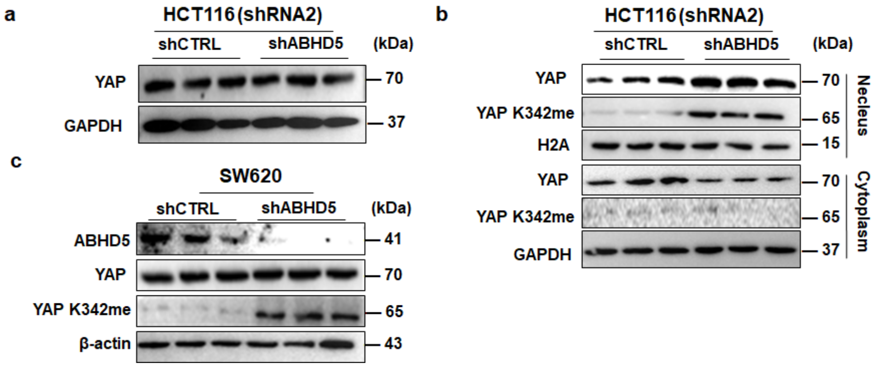
a
HCT116(shRNA2)
shCTRL
shABHD5
(kDa)
YAP
70
GAPDH
37
b
HCT116(shRNA2)
shCTRL
shABHD5
(kDa)
YAP
70
YAP K342me
65
H2A
15
Necleus
YAP
70
YAP K342me
65
GAPDH
37
Cytoplasm
c
SW620
shCTRL
shABHD5
(kDa)
ABHD5
41
YAP
70
YAP K342me
65
β-actin
43

Fig S7

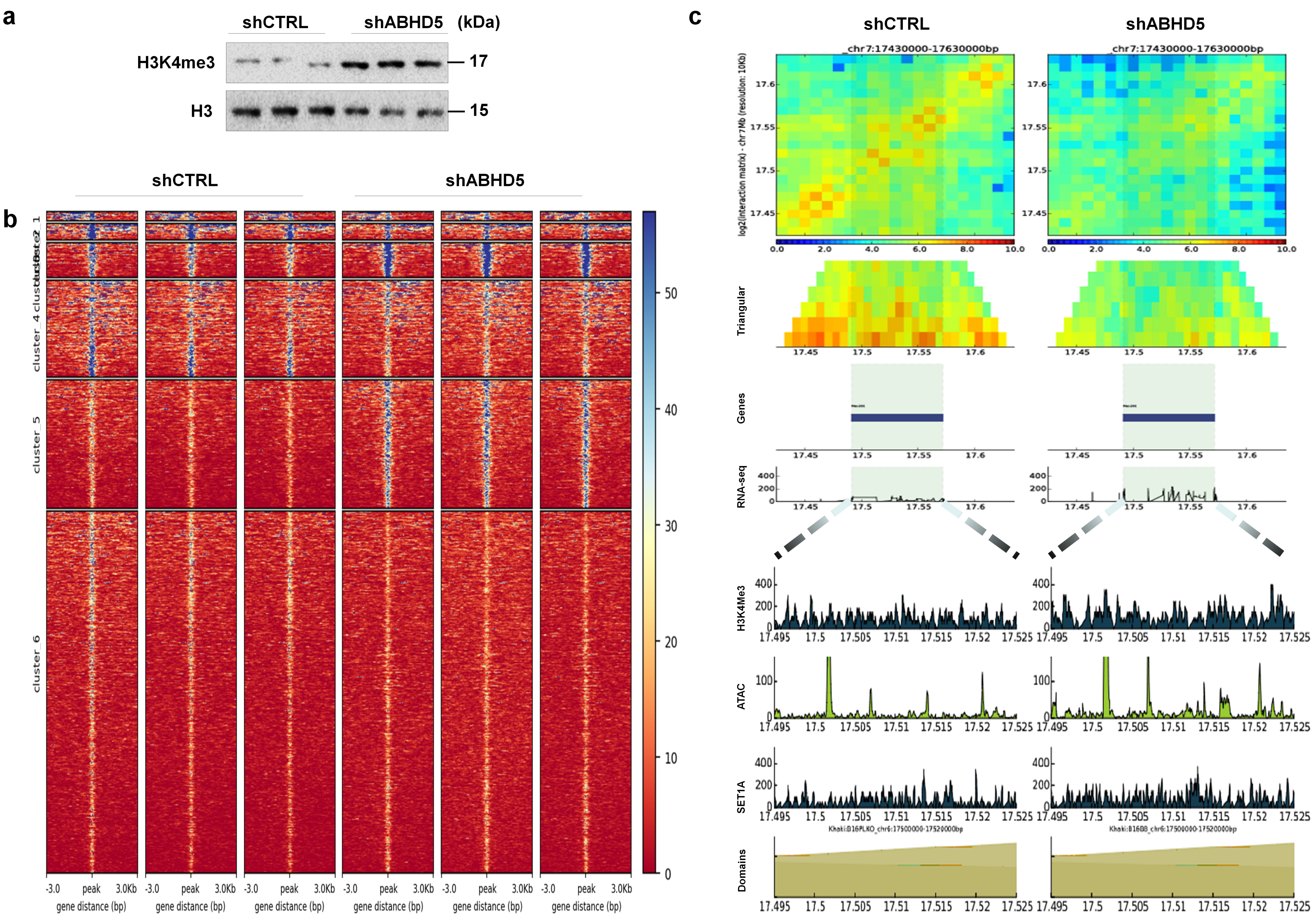

a
shCTRL
shABHD5
(kDa)
H3K4me3
17
H3
15
b
shCTRL
shABHD5
cluster_1
cluster_4
cluster_5
cluster_6
50
40
30
20
10
0
-3.0
peak
3.0Kb
gene distance (bp)
c
shCTRL
shABHD5
chr7:17430000-17630000bp
log2(interaction matrix) - chr7Mb (resolution: 10Kb)
Triangular
Genes
RNA-seq
H3K4Me3
ATAC
SET1A
Domains
17.495
17.5
17.505
17.51
17.515
17.52
17.525

Fig S8

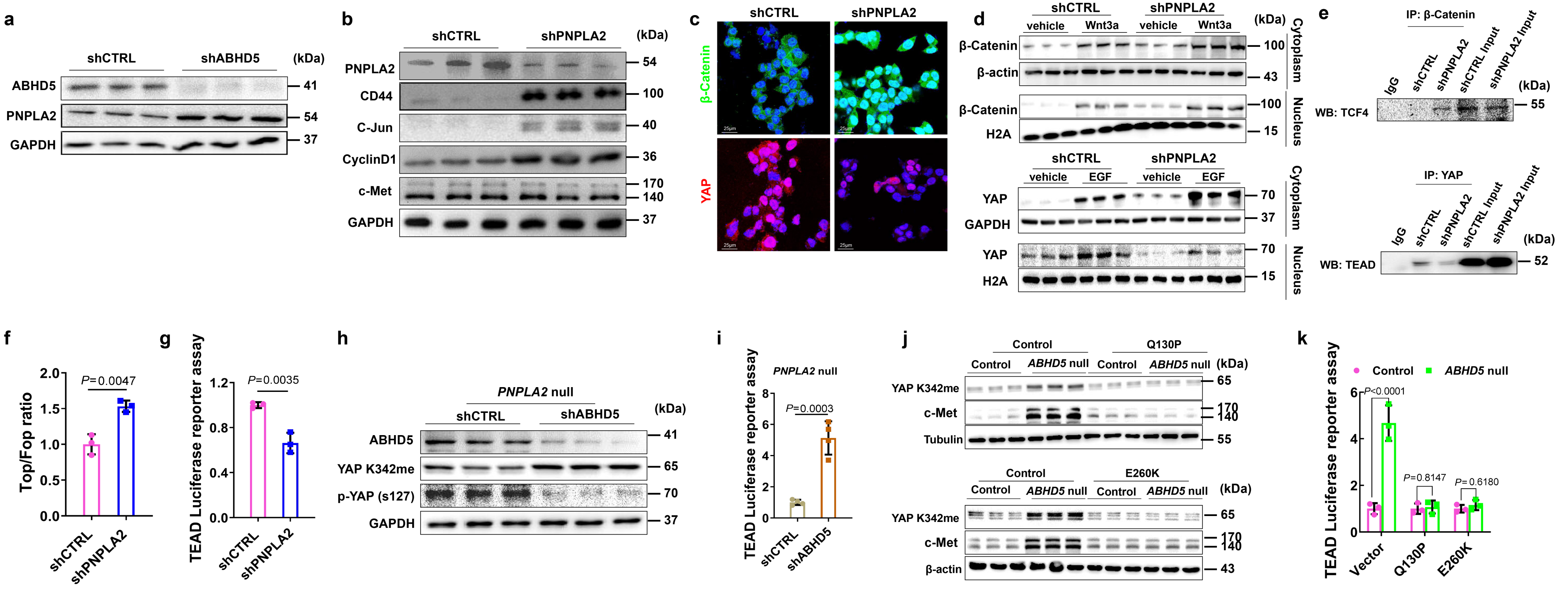
a
shCTRL
shABHD5
(kDa)
ABHD5
PNPLA2
GAPDH
41
54
37
b
shCTRL
shPNPLA2
(kDa)
PNPLA2
CD44
C-Jun
CyclinD1
c-Met
GAPDH
54
100
40
36
170
140
37
c
shCTRL
shPNPLA2
β-Catenin
YAP
d
shCTRL
shPNPLA2
vehicle
Wnt3a
(kDa)
β-Catenin
β-actin
H2A
Cytoplasm
Nucleus
100
43
15
EGF
YAP
GAPDH
70
37
e
IP: β-Catenin
IgG
shCTRL
shPNPLA2
shCTRL Input
shPNPLA2 Input
WB: TCF4
55
IP: YAP
WB: TEAD
52
f
Top/Fop ratio
P=0.0047
g
TEAD Luciferase reporter assay
P=0.0035
h
PNPLA2 null
YAP K342me
p-YAP (s127)
65
i
PNPLA2 null
P=0.0003
shABHD5
j
Control
Q130P
ABHD5 null
Tubulin
55
E260K
k
Control
ABHD5 null
P<0.0001
P=0.8147
P=0.6180
Vector
Q130P
E260K

Fig S9

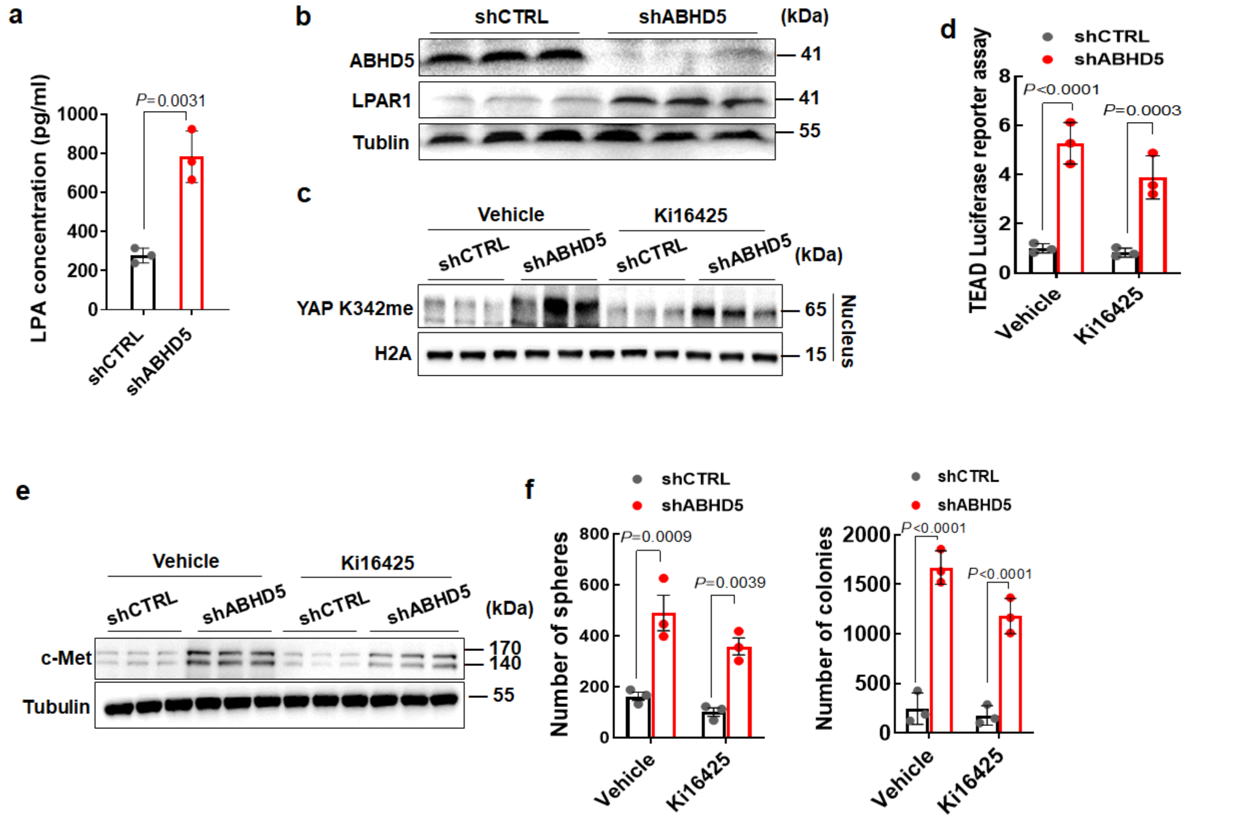

a
LPA concentration (pg/ml)
P=0.0031
shCTRL
shABHD5
b
shCTRL
shABHD5
(kDa)
ABHD5
LPAR1
Tublin
41
41
55
c
Vehicle
Ki16425
shCTRL
shABHD5
shCTRL
shABHD5
(kDa)
YAP K342me
H2A
65
15
Nucleus
d
shCTRL
shABHD5
TEAD Luciferase reporter assay
P<0.0001
P=0.0003
Vehicle
Ki16425
e
Vehicle
Ki16425
shCTRL
shABHD5
shCTRL
shABHD5
(kDa)
c-Met
Tubulin
170
140
55
f
shCTRL
shABHD5
Number of spheres
P=0.0009
P=0.0039
Vehicle
Ki16425
Number of colonies
P<0.0001
P<0.0001
Vehicle
Ki16425

Fig S10

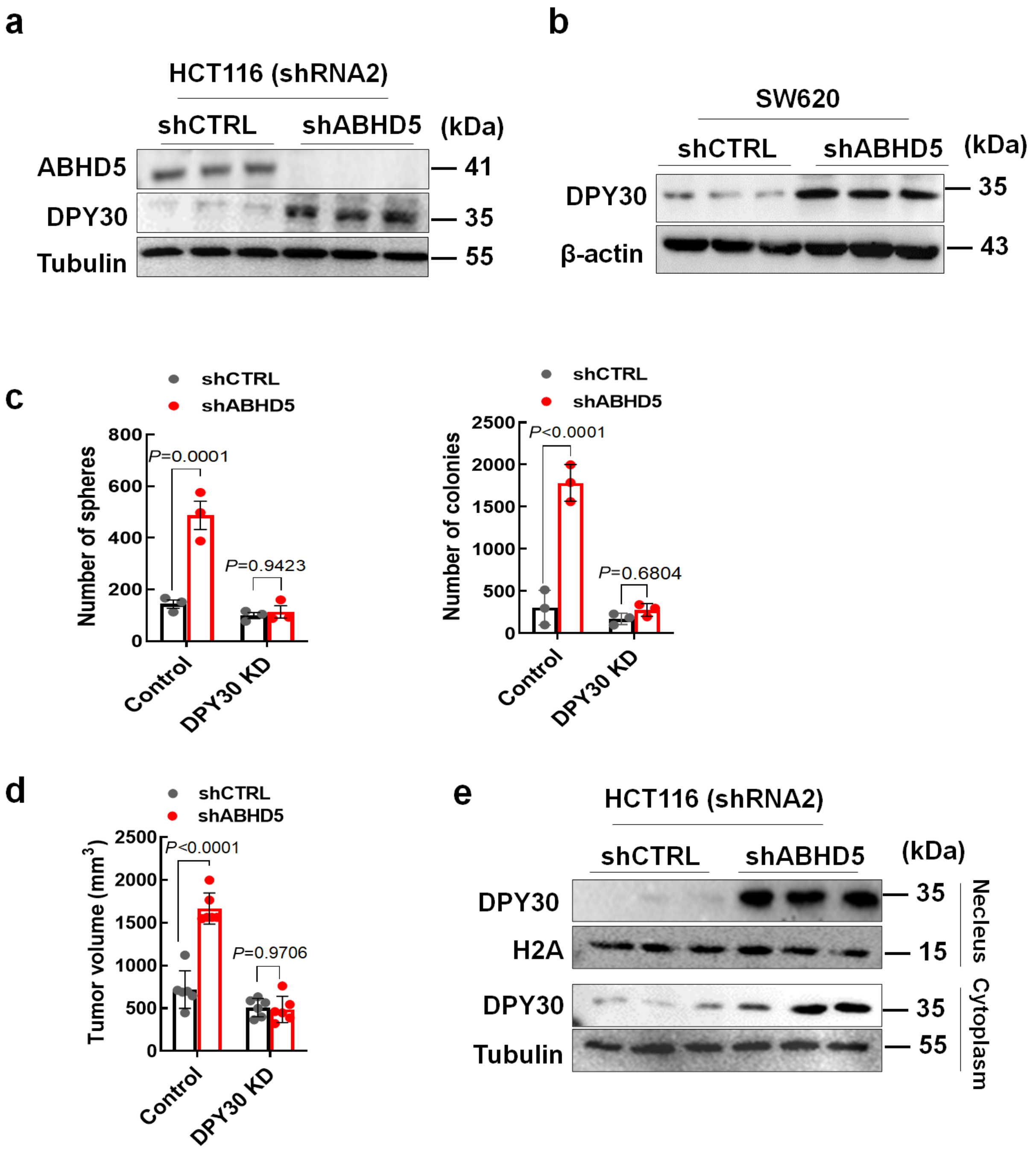

a
HCT116 (shRNA2)
shCTRL
shABHD5
(kDa)
ABHD5
41
DPY30
35
Tubulin
55
b
SW620
shCTRL
shABHD5
(kDa)
DPY30
35
β-actin
43
c
shCTRL
shABHD5
Number of spheres
800
600
400
200
0
P=0.0001
P=0.9423
Control
DPY30 KD
shCTRL
shABHD5
Number of colonies
2500
2000
1500
1000
500
0
P<0.0001
P=0.6804
Control
DPY30 KD
d
shCTRL
shABHD5
Tumor volume (mm3)
2500
2000
1500
1000
500
0
P<0.0001
P=0.9706
Control
DPY30 KD
e
HCT116 (shRNA2)
shCTRL
shABHD5
(kDa)
DPY30
H2A
DPY30
Tubulin
35
15
35
55
Necleus
Cytoplasm

Fig S11

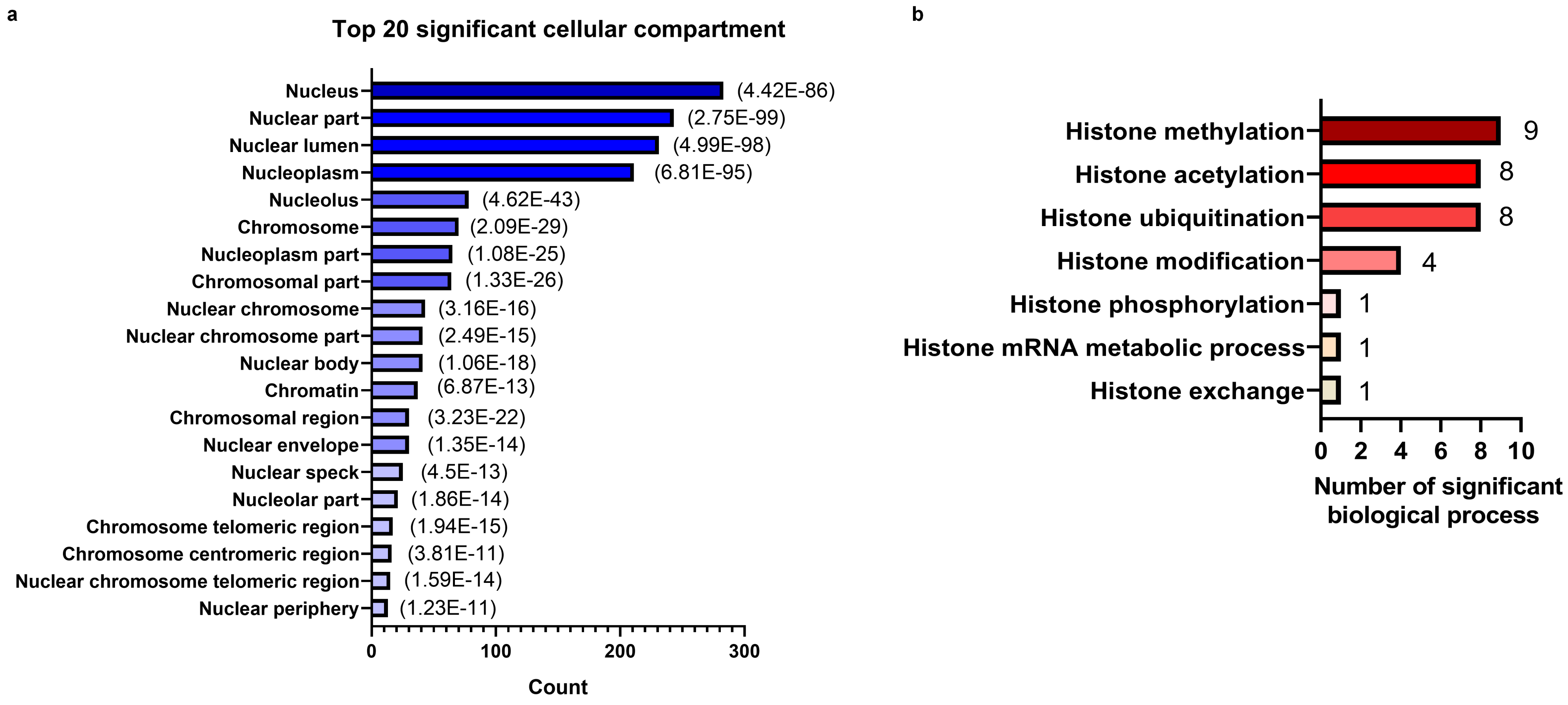
a
Top 20 significant cellular compartment
Nucleus (4.42E-86)
Nuclear part (2.75E-99)
Nuclear lumen (4.99E-98)
Nucleoplasm (6.81E-95)
Nucleolus (4.62E-43)
Chromosome (2.09E-29)
Nucleoplasm part (1.08E-25)
Chromosomal part (1.33E-26)
Nuclear chromosome (3.16E-16)
Nuclear chromosome part (2.49E-15)
Nuclear body (1.06E-18)
Chromatin (6.87E-13)
Chromosomal region (3.23E-22)
Nuclear envelope (1.35E-14)
Nuclear speck (4.5E-13)
Nucleolar part (1.86E-14)
Chromosome telomeric region (1.94E-15)
Chromosome centromeric region (3.81E-11)
Nuclear chromosome telomeric region (1.59E-14)
Nuclear periphery (1.23E-11)
0
100
200
300
Count
b
Histone methylation 9
Histone acetylation 8
Histone ubiquitination 8
Histone modification 4
Histone phosphorylation 1
Histone mRNA metabolic process 1
Histone exchange 1
0 2 4 6 8 10
Number of significant biological process
