# Supplementary material for: ABHD5 inhibits YAP-induced c-Met overexpression and colon cancer cell stemness via suppressing YAP methylation
Source: Nat Commun. 2021 Nov 18;12:6711. doi: 10.1038/s41467-021-26967-5 (PMC8602706; doi:10.1038/s41467-021-26967-5)
Supplement: Supplementary file 3 — Reporting Summary [file 41467_2021_26967_MOESM3_ESM.pdf]

nature research

Corresponding author(s): Juanjuan Ou

Last updated by author(s): Oct 3, 2021

Reporting Summary

Nature Research wishes to improve the reproducibility of the work that we publish. This form provides structure for consistency and transparency in reporting. For further information on Nature Research policies, see our Editorial Policies and the Editorial Policy Checklist.

Statistics

For all statistical analyses, confirm that the following items are present in the figure legend, table legend, main text, or Methods section.

n/a	Confirmed	
☐	☒	The exact sample size (*n*) for each experimental group/condition, given as a discrete number and unit of measurement
☐	☒	A statement on whether measurements were taken from distinct samples or whether the same sample was measured repeatedly
☐	☒	The statistical test(s) used AND whether they are one- or two-sided<br>*Only common tests should be described solely by name; describe more complex techniques in the Methods section.*
☐	☒	A description of all covariates tested
☐	☒	A description of any assumptions or corrections, such as tests of normality and adjustment for multiple comparisons
☐	☒	A full description of the statistical parameters including central tendency (e.g. means) or other basic estimates (e.g. regression coefficient) AND variation (e.g. standard deviation) or associated estimates of uncertainty (e.g. confidence intervals)
☐	☒	For null hypothesis testing, the test statistic (e.g. *F*, *t*, *r*) with confidence intervals, effect sizes, degrees of freedom and *P* value noted<br>*Give P values as exact values whenever suitable.*
☒	☐	For Bayesian analysis, information on the choice of priors and Markov chain Monte Carlo settings
☒	☐	For hierarchical and complex designs, identification of the appropriate level for tests and full reporting of outcomes
☐	☒	Estimates of effect sizes (e.g. Cohen's *d*, Pearson's *r*), indicating how they were calculated

Our web collection on statistics for biologists contains articles on many of the points above.

Software and code

Policy information about availability of computer code

Data collection	BD LSRII flow-cytometer was used to collect flow cytometry data.
Data analysis	FACSDiva (v8.0.1) software (Bekton Dikinson) for FACS results.<br>ImageJ (v1.8.0) (NIH, Bethesda, MD) for pictures.<br>GraphPad Prism 8 for statistics.<br>L-Calc software for stem cell frequency analysis.

For manuscripts utilizing custom algorithms or software that are central to the research but not yet described in published literature, software must be made available to editors and reviewers. We strongly encourage code deposition in a community repository (e.g. GitHub). See the Nature Research guidelines for submitting code & software for further information.

Data

Policy information about availability of data

All manuscripts must include a data availability statement. This statement should provide the following information, where applicable:

- Accession codes, unique identifiers, or web links for publicly available datasets
- A list of figures that have associated raw data
- A description of any restrictions on data availability

The processed sequencing data used in this study is available at the gene expression omnibus (GEO) database under accession code GSE185056(https://www.ncbi.nlm.nih.gov/geo/query/acc.cgi?acc=GSE185056).The data that support the findings of this study are available within the article and its Supplementary Information files or from the corresponding author upon reasonable request. Source data are provided with this paper.

Field-specific reporting

Please select the one below that is the best fit for your research. If you are not sure, read the appropriate sections before making your selection.

- [x] Life sciences
- [ ] Behavioural & social sciences
- [ ] Ecological, evolutionary & environmental sciences

For a reference copy of the document with all sections, see nature.com/documents/nr-reporting-summary-flat.pdf

Life sciences study design

All studies must disclose on these points even when the disclosure is negative.

Sample size	The minimal sample size n of cell and animal experiments were determined by explanatory variable k , n>=k+1. The human colon cancer tissue microarray purchased from Shanghai Outdo Biotech Co.,ltd. with 107 samples were used for correlation and survival analyses. The surgically resected tissues from two CRC patients for ex vivo sphere formation assay were collected from Fuling Central Hospital. Sample size was chosen based on our previous published work and preliminary studies.
Data exclusions	No data were excluded.
Replication	All the experimental findings were reliably reproduced as validated by independent experiments and biological replicates. Data presented in the manuscript was performed using at least N=3 biological replicates.
Randomization	Age- and sex-matched mice were assigned randomly to experimental and control groups. For both in vitro and in vivo experiments, cells were randomly assigned to experimental groups, or based on genotype and treatment conditions. The human colon cancer tissue microarray samples were grouped based on genotype. No additional randomization was applicable to the study.
Blinding	The investigators were not blinded to group allocation during data collection or analysis. Animal treatments, in vitro and in vivo experiments were performed by technicians who were not blind, but they didn't participated in sample measurement and analysis. The principal investigators were not blind, because they had their own independent projects, and it is impossible for others to replace them to collect and analyze.

Reporting for specific materials, systems and methods

We require information from authors about some types of materials, experimental systems and methods used in many studies. Here, indicate whether each material, system or method listed is relevant to your study. If you are not sure if a list item applies to your research, read the appropriate section before selecting a response.

Materials & experimental systems

n/a	Involved in the study	
[ ]	[x]	Antibodies
[ ]	[x]	Eukaryotic cell lines
[x]	[ ]	Palaeontology and archaeology
[ ]	[x]	Animals and other organisms
[x]	[ ]	Human research participants
[x]	[ ]	Clinical data
[x]	[ ]	Dual use research of concern

Methods

n/a	Involved in the study	
[ ]	[x]	ChIP-seq
[ ]	[x]	Flow cytometry
[x]	[ ]	MRI-based neuroimaging

Antibodies

Antibodies used

ABHD5 [NOVUS,H00051099-M01,RRID:AB_536536 (for WB,IHC),Mouse,Dilution(WB 1:1000;IHC 1:250)]
ABHD5 [SIGMA,HPA035851,RRID:AB_10671942 (for IF),Rabbit,Dilution(IHC 1:200)]
β-Actin [EarthOx,E021070-01,Rabbit,Dilution(WB 1:1000)]
c-Met [CST,8198,RRID:AB_10858224 (for WB),Rabbit,Dilution(WB 1:1000)]
c-Met [Santa,SC-8057,RRID:AB_673755 (for IHC),Mouse,Dilution(IHC 1:200)]
GAPDH [Proteintech,60004-1-lg,RRID:AB_2107436,Mouse,Dilution(WB 1:20000)]
β-Catenin [Santa,SC-393501 (IHC),Mouse,Dilution(IHC 1:200)]
β-Catenin [CST,8480,RRID:AB_11127855 (for WB,IF),Rabbit,Dilution(WB 1:1000;IF 1:100)]
TCF4 [Santa,SC-166699,RRID:AB_2199823(for WB),Mouse,Dilution(WB 1:1000)]
TCF4 [CST,2569,RRID:AB_2199816 (for CHIP),Rabbit,Dilution(CHIP 1:50)]
YAP1 [NOVUS,NB110-58358,RRID:AB_922796 (for WB,IF and CHIP),Rabbit,Dilution(WB 1:1000;IF 1:500;CHIP 1:200)]
YAP1 [Santa,SC-101199,RRID:AB_1131430 (for IHC),Mouse,Dilution(IHC 1:200)]
YAP1 (SU33-06) [NOVUS,NBP2-67467(for IP),Rabbit,Dilution(IP 1:200)]
H2A [NOVUS,NB100-56346,RRID:AB_838346,Rabbit,Dilution(WB 1:1000)]

TEAD1 [Abcam,Ab133533,RRID:AB_2737294,,Dilution()]
Tubulin [Proteintech,HRP-66031,RRID:AB_2687491,Mouse,Dilution(WB 1:5000)]
GFP [NOVUS,NB600-308,RRID:AB_10003058,Rabbit,Dilution(WB 1:1000)]
YAP km342 [College of Life Science and Technology of Tongji University,Not applicable,Rabbit,Dilution(WB 1:1000)]
p-YAP(S127) [Abcam,Ab205270,RRID:AB_2813833,Rabbit,Dilution(WB 1:1000)]
CRM1(A-7) [Santa,SC-374124,RRID:AB_10917075,Mouse,Dilution(WB 1:1000)]
ATGL(PNPLA2) [CST,2439,Rabbit,Dilution(WB 1:1000)]
CD44 [CST,3570,RRID:AB_2076465,Mouse,Dilution(F 1:100)]
C-Jun [CST,9165,Rabbit,Dilution(WB 1:1000)]
CyclinD1 [CST,2978,Rabbit,Dilution(WB 1:1000)]
DPY30 [NOVUS,45110002,RRID:AB_10702185 (for WB),Rabbit,Dilution(WB 1:1000)]
DPY30 [NOVUS,NBP2-32201 (for IHC),Rabbit,Dilution(IHC 1:1000)]
Ubiquitin [Abcam,Ab134953,RRID:AB_2801561,Rabbit,Dilution(WB 1:1000)]
LPAR1 [Abcam,Ab23698,RRID:AB_447619,Rabbit,Dilution(WB 1:1000)]
The Alexa Fluor 594 anti-mouse IgG [Thermo Fisher,A11037,Rabbit,Dilution(IF 1:400)]
The Alexa Fluor 647 anti-mouse IgG [Santa,sc-516609,Mouse,Dilution(IF 1:200)]
The FITC anti-rabbit IgG [EarthOx,E031220,Goat,Dilution(IF 1:200)]
The dylight 649 anti-Rabbit IgG(H+L) [EarthOx,E032620,Goat,Dilution(IF 1:200)]
Anti-histone H3(trimethyl K4) [Abcam,Ab8580,RRID:AB_306649(for Chip, WB),Rabbit,Dilution(WB 1:1000)]
Anti-hSET1/SET [Abcam,Ab70378,RRID:AB_1951955(for Chip,WB),Rabbit,Dilution(WB 1:5000)]
SETD1A [Novus,NB100-558,RRID:AB_2185760(for IP, WB and Chip),Rabbit,Dilution(IP 1:200;WB 1:10000;CHIP 1： 200)]
PE/Cy7 anti-mouse/human CD44 [Biolegend,103029,Rat,Dilution(Not applicable)]
APC anti-Human CD133 [BD,560596,Mouse,Dilution(Not applicable)]
Lgr5/GPR49 Antibody [novus,NLS1236,, RRID:AB_10001136,Rabbit,Dilution(IHC 1:200)]

Validation

ABHD5 [NOVUS,H00051099-M01,RRID:AB_536536 (for WB,IHC),Mouse,Dilution(WB 1:1000;IHC 1:250) https://www.novusbio.com/products/abhd5-antibody-1f3_h00051099-m01]
ABHD5 [SIGMA,HPA035851,RRID:AB_10671942 (for IF),Rabbit,Dilution(IHC 1:200) https://www.sigmaaldrich.cn/CN/zh/product/sigma/hpa035851?context=product#]
β-Actin [EarthOx,E021070-01,Rabbit,Dilution(WB 1:1000) https://earthox.net/product/anti-%ce%b2-actin-rabbit-polyclonal-antibody/]
c-Met [CST,8198,RRID:AB_10858224 (for WB),Rabbit,Dilution(WB 1:1000) https://www.cellsignal.cn/products/primary-antibodies/met-d1c2-xp-rabbit-mab/8198?site-search-type=Products&N=4294956287&Ntt=8198&fromPage=plp&_requestid=422410]
c-Met [Santa,SC-8057,RRID:AB_673755 (for IHC),Mouse,Dilution(IHC 1:200) https://www.scbt.com/p/met-antibody-b-2?requestFrom=search]
GAPDH [Proteintech,60004-1-Ig,RRID:AB_2107436,Mouse,Dilution(WB 1:20000) https://www.ptgcn.com/products/GAPDH-Antibody-60004-1-Ig.htm]
β-Catenin [Santa,SC-393501 (IHC),Mouse,Dilution(IHC 1:200) https://www.scbt.com/p/beta-catenin-antibody-a-5?requestFrom=search]
β-Catenin [CST,8480,RRID:AB_11127855 (for WB,IF),Rabbit,Dilution(WB 1:1000;IF 1:100) https://www.cellsignal.cn/products/primary-antibodies/b-catenin-d10a8-xp-rabbit-mab/8480?site-search-type=Products&N=4294956287&Ntt=8480&fromPage=plp&_requestid=422527]
TCF4 [Santa,SC-166699,RRID:AB_2199823(for WB),Mouse,Dilution(WB 1:1000) https://www.scbt.com/p/tcf-4-antibody-d-4?requestFrom=search]
TCF4 [CST,2569,RRID:AB_2199816 (for CHIP),Rabbit,Dilution(CHIP 1:50) https://www.cellsignal.cn/products/primary-antibodies/tcf4-tcf7l2-c48h11-rabbit-mab/2569?site-search-type=Products&N=4294956287&Ntt=2569&fromPage=plp&_requestid=422695]
YAP1 [NOVUS,NB110-58358,RRID:AB_922796 (for WB,IF and CHIP),Rabbit,Dilution(WB 1:1000;IF 1:500;CHIP 1:200) https://www.novusbio.com/products/yap1-antibody_nb110-58358]
YAP1 [Santa,SC-101199,RRID:AB_1131430 (for IHC),Mouse,Dilution(IHC 1:200) https://www.scbt.com/p/yap-antibody-63-7?requestFrom=search]
YAP1 (SU33-06) [NOVUS,NBP2-67467(for IP),Rabbit,Dilution(IP 1:200) https://www.novusbio.com/products/yap1-antibody-su33-06_nbp2-67467]
H2A [NOVUS,NB100-56346,RRID:AB_838346,Rabbit,Dilution(WB 1:1000) https://www.novusbio.com/products/histone-h2a-antibody_nb100-56346]
TEAD1 [Abcam,Ab133533,RRID:AB_2737294,,Dilution()]
Tubulin [Proteintech,HRP-66031,RRID:AB_2687491,Mouse,Dilution(WB 1:5000) https://www.ptgcn.com/products/Tubulin-Alpha-Antibody-HRP-66031.htm]
GFP [NOVUS,NB600-308,RRID:AB_10003058,Rabbit,Dilution(WB 1:1000) https://www.novusbio.com/products/gfp-antibody_nb600-308]
YAP km342 [College of Life Science and Technology of Tongji University,Not applicable,Rabbit,Dilution(WB 1:1000) https://www.cell.com/cancer-cell/fulltext/S1535-6108(18)30230-7]
p-YAP(S127) [Abcam,Ab205270,RRID:AB_2813833,Rabbit,Dilution(WB 1:1000) https://www.abcam.cn/active-yap1-antibody-epr19812-ab205270.html]
CRM1(A-7) [Santa,SC-374124,RRID:AB_10917075,Mouse,Dilution(WB 1:1000) https://www.scbt.com/p/crm1-antibody-a-7?requestFrom=search]
ATGL(PNPLA2) [CST,2439,Rabbit,Dilution(WB 1:1000) https://www.cellsignal.cn/products/primary-antibodies/atgl-30a4-rabbit-mab/2439?site-search-type=Products&N=4294956287&Ntt=atgl&fromPage=plp]
CD44 [CST,3570,RRID:AB_2076465,Mouse,Dilution(F 1:100) https://www.cellsignal.cn/products/primary-antibodies/cd44-156-3c11-mouse-mab/3570?site-search-type=Products&N=4294956287&Ntt=3570&fromPage=plp&_requestid=423284]
C-Jun [CST,9165,Rabbit,Dilution(WB 1:1000) https://www.cellsignal.cn/products/primary-antibodies/c-jun-60a8-rabbit-mab/9165?

site-search-type=Products&N=4294956287&Ntt=9165&fromPage=plp&_requestid=423462]
CyclinD1 [CST,2978,Rabbit,Dilution(WB 1:1000) https://www.cellsignal.cn/products/primary-antibodies/cyclin-d1-92g2-rabbit-mab/2978?site-search-type=Products&N=4294956287&Ntt=2978&fromPage=plp&_requestid=423579]
DPY30 [NOVUS,45110002,RRID:AB_10702185 (for WB),Rabbit,Dilution(WB 1:1000) https://www.novusbio.com/products/dpy30-antibody_45110002]
DPY30 [NOVUS,NBP2-32201 (for IHC),Rabbit,Dilution(IHC 1:1000) https://www.novusbio.com/products/dpy30-antibody_nbp2-32201]
Ubiquitin [Abcam,Ab134953,RRID:AB_2801561,Rabbit,Dilution(WB 1:1000) https://www.abcam.cn/ubiquitin-antibody-epr8830-ab134953.html]
LPAR1 [Abcam,Ab23698,RRID:AB_447619,Rabbit,Dilution(WB 1:1000) https://www.abcam.cn/edg2-lpa-1-antibody-ab23698.html]
The Alexa Fluor 594 anti-mouse IgG [Thermo Fisher,A11037,Rabbit,Dilution(IF 1:400) https://www.thermofisher.cn/cn/zh/antibody/product/Goat-anti-Rabbit-IgG-H-L-Highly-Cross-Adsorbed-Secondary-Antibody-Polyclonal/A-11037]
The Alexa Fluor 647 anti-mouse IgG [Santa,sc-516609,Mouse,Dilution(IF 1:200) https://www.scbt.com/p/normal-mouse-igg-alexa-fluor-647?requestFrom=search]
The FITC anti-rabbit IgG [EarthOx,E031220,Goat,Dilution(IF 1:200) https://earthox.net/product/fitc-goat-anti-rabbit-igghl/]
The dylight 649 anti-Rabbit IgG(H+L) [EarthOx,E032620,Goat,Dilution(IF 1:200) http://www.canlifesci.com/Product/Detail.aspx?Id=6210]
Anti-histone H3(trimethyl K4) [Abcam,Ab8580,RRID:AB_306649(for Chip, WB),Rabbit,Dilution(WB 1:1000) https://www.cellsignal.cn/products/primary-antibodies/cyclin-d1-92g2-rabbit-mab/2978?site-search-type=Products&N=4294956287&Ntt=2978&fromPage=plp&_requestid=423579]
Anti-hSET1/SET [Abcam,Ab70378,RRID:AB_1951955(for Chip,WB),Rabbit,Dilution(WB 1:5000) https://www.abcam.cn/hset1set1-antibody-ab70378.html]
SETD1A [Novus,NB100-558,RRID:AB_2185760(for IP, WB and Chip),Rabbit,Dilution(IP 1:200;WB 1:10000;CHIP 1：200) https://www.novusbio.com/products/setd1a-antibody_nb100-558]
PE/Cy7 anti-mouse/human CD44 [Biolegend,103029,Rat,Dilution(Not applicable) https://www.biolegend.com/en-us/search-results/pe-cyanine7-anti-mouse-human-cd44-antibody-3932]
APC anti-Human CD133 [BD,560596,Mouse,Dilution(Not applicable) https://www.bdbiosciences.com/zh-cn/products/reagents/flow-cytometry-reagents/research-reagents/single-color-antibodies-ruo/apc-mouse-anti-human-cd133.566596]
Lgr5/GPR49 Antibody [novus,NLS1236,, RRID:AB_10001136,Rabbit,Dilution(IHC 1:200) https://www.novusbio.com/products/lgr5-gpr49-antibody_nls1236]

Eukaryotic cell lines

Policy information about cell lines

Cell line source(s)	HCT 116, RKO, SW620 and CT26 cell lines were purchased from ATCC.MC38 cell lines were purchased from Fuheng BioLogy Corporation.
Authentication	STR by source.
Mycoplasma contamination	There is no mycoplasma contamination in all these cell lines.
Commonly misidentified lines (See ICLAC register)	There is no commonly misdentified lines in all these cell lines.

Animals and other organisms

Policy information about studies involving animals; ARRIVE guidelines recommended for reporting animal research

Laboratory animals	Mice were housed and bred at the Medical Research Center of Southwest Hospital in specific pathogen-free conditions. All animals were housed under a controlled temperature (22±2°C), humidity (55±5%) and a 12 light-dark cycle (light on 7 am) with free access to food and water.<br><br>Both male and female mice were used for analysis and quantification.<br><br>Intestine-specific Abhd5-knockout mice were generated by mating Abhd5-floxed mice created by Cyagen Biosciences (Suzhou) Inc. with B6.Cg-Tg (Vil1-cre) 977Gum/J mice (Jackson Laboratory, stock #004586). A male ApcMin/+ mouse on the C57BL/6J background was purchased from The Jackson Laboratory (stock #002020) and crossed with female intestine-specific Abhd5-knockout mice to produce ApcMin/+ mice lacking Abhd5 in the intestine and their control littermates for experiments.<br><br>Six- to eight-week-old male NOD/SCID mice on the C57BL/6J background were purchased from the Chinese Academy of Sciences Shanghai SLAC Laboratory Animal Co. (SLACCAS, Shanghai, China) and acclimated for 4 days.
Wild animals	We have not used wild animals in this study.
Field-collected samples	No field collected samples were used in the study
Ethics oversight	All animal experiments were approved by the Institutional Animal Care and Use Committee of the Third Military Medical University (Army Medical University) in accordance with the Guide for the Care and Use of Laboratory Animals.

Note that full information on the approval of the study protocol must also be provided in the manuscript.

ChIP-seq

Data deposition

☐ Confirm that both raw and final processed data have been deposited in a public database such as GEO.

☐ Confirm that you have deposited or provided access to graph files (e.g. BED files) for the called peaks.

Data access links<br>*May remain private before publication.*	https://pan.baidu.com/s/1krGEdzh4iLGnYu9sTy9vIw<br>Access code: b805
Files in database submission	*Provide a list of all files available in the database submission.*
Genome browser session<br>(e.g. UCSC)	*Provide a link to an anonymized genome browser session for "Initial submission" and "Revised version" documents only, to enable peer review. Write "no longer applicable" for "Final submission" documents.*

Methodology

Replicates	*Describe the experimental replicates, specifying number, type and replicate agreement.*
Sequencing depth	*Describe the sequencing depth for each experiment, providing the total number of reads, uniquely mapped reads, length of reads and whether they were paired- or single-end.*
Antibodies	We used anti- SET1A, YAP, H3K4me3 antibodies
Peak calling parameters	*Specify the command line program and parameters used for read mapping and peak calling, including the ChIP, control and index files used.*
Data quality	*Describe the methods used to ensure data quality in full detail, including how many peaks are at FDR 5% and above 5-fold enrichment.*
Software	*Describe the software used to collect and analyze the ChIP-seq data. For custom code that has been deposited into a community repository, provide accession details.*

Flow Cytometry

Plots

Confirm that:

☒ The axis labels state the marker and fluorochrome used (e.g. CD4-FITC).

☒ The axis scales are clearly visible. Include numbers along axes only for bottom left plot of group (a 'group' is an analysis of identical markers).

☒ All plots are contour plots with outliers or pseudocolor plots.

☒ A numerical value for number of cells or percentage (with statistics) is provided.

Methodology

Sample preparation	HCT116 colon cancer cells were stained using APC-conjugated CD133 (BD Biosciences) and PE-Cy7-conjugated CD44 (BioLegend). Samples were analyzed on a BD LSRII flow-cytometer (Bekton Dikinson, Franklin Lakes, NJ, USA). Analysis of cytometric data was performed using FACSDiva software (Bekton Dikinson).<br>For the identification of ALDH+ cells, the ALDEFLUOR kit was used to sort ALDH+ cells with high ALDH enzymatic activity. Single-cell suspensions were made in ALDH assay buffer containing the ALDH substrate-BAAA (BODIPY-aminoacetaldehyde, 1mmol/L/1x106 cells) and then incubated for 40 min at 37°C. In each experiment, the specific ALDH inhibitor diethylaminobenzaldehyde (DEAB) was used as a control at a concentration of 15 mmol/L. The specific ALDH activity was calculated according to the difference in activity between the presence and absence of the inhibitor DEAB.
Instrument	BD LSRII flow-cytometer (Bekton Dikinson, Franklin Lakes, NJ, USA).
Software	FACSDiva software (Bekton Dikinson).
Cell population abundance	The purities of the sorted cells were more than 98%.
Gating strategy	For all experiments FSC-A vs. SSC-A gates of the starting cell population were used to identify viable cells. Singlet cells were identified using FSC-A vs. FSC-H gating. Positive populations were determined by the specific antibodies, which were distinct from negative populations.

☒ Tick this box to confirm that a figure exemplifying the gating strategy is provided in the Supplementary Information.
